# Promoting the adoption of behaviors to prevent osteoporosis using the health belief model integrated with health literacy: quasi-experimental intervention study

**DOI:** 10.1186/s12889-021-12300-8

**Published:** 2021-12-07

**Authors:** Rahman Panahi, Fatemeh Samiei Siboni, Mansoure Kheiri, Khadije Jahangasht Ghoozlu, Mahya Shafaei, Leila Dehghankar

**Affiliations:** 1 Department of Health Education & Promotion, School of Medical Sciences, Tarbiat Modares University, Tehran, Iran; 2 Department of Nursing, Social Determinants of Health Research Center, Research Institute for Prevention of Non-Communicable Diseases, School of Nursing & Midwifery, Qazvin University of Medical Sciences, Qazvin, Iran; 3 School of Nursing and Midwifery, Shahroud University of Medical Sciences, Shahroud, Iran; 4 Ramsar School of Nursing, Babol University of Medical Sciences, Babol, Iran

**Keywords:** Health belief model, Health literacy, Osteoporosis, Educational intervention

## Abstract

**Background:**

The health belief model (HBM) is effective in preventing osteoporosis and promoting health literacy (HL). In this regard, there are some critical points such as the role of HL in preventing osteoporosis, adoption of preventive behaviors, adoption of behaviors, including physical activity, and the role of health volunteers in transmitting health messages to the community. Considering the aforesaid points this study was performed among the health volunteers aimed to determine the effect of educational intervention based on integrated HBM with HL on walking and nutrition behaviors to prevent osteoporosis.

**Materials and methods:**

In this quasi-experimental intervention study, 140 health volunteers (70 people in each of the two intervention and control groups) were enrolled in the study using multi-stage random sampling, in 2020. The members of the intervention group received e-learning through social media software, 4 times during 4 weeks (once a week) and were provided with educational booklets and pamphlets. Data collection tools included demographic and background questionnaires; standard questionnaire based on the HBM, awareness, and walking and nutrition behaviors to prevent osteoporosis; and HELIA questionnaire to measure HL. These questionnaires were completed in two stages, before and 3 months after the intervention. The educational intervention in this study was sent to the intervention group in 4 stages. The collected data were analyzed using proportional tests (paired t-test, Wilcoxon test, independent t-test, Mann-Whitney test) and SPSS software version 23.

**Results:**

The mean and standard deviation related to the score of adoption of nutrition behaviors at the beginning of the study in the intervention group was 5.398 ± 1.447, which changed to 8.446 ± 1.244 after 3 months, indicating a significant increase in the adoption of such behaviors (*P* = 0.009). In the control group, the mean and standard deviation of the scores of adoption of nutrition behaviors changed from 5.451 ± 1.222 to 6.003 ± 1.005, which was not statistically significant (*P* = 0.351). Also, the mean and standard deviation related to the scores of adoption of walking behavior at the beginning of the study in the intervention group was 8.956 ± 0.261, which changed to 13.457 ± 0.587 after 3 months, indicating a significant increase in the adoption of such behaviors (*P* < 0.001). In the control group, the mean and standard deviation related to the scores of the adoption of walking behavior changed from 8.848 ± 0.353 to 9.025 ± 0.545, which was not statistically significant (*P* = 0.211).

Prior to the intervention, there was no significant difference between two groups regarding the variables of demographic and background, knowledge, all constructs of the model, HL, and adoption of walking and nutrition behaviors (*P* > 0.05). After the intervention, the comparison of the two groups showed that there was a significant change in the mean scores of awareness, all constructs of the model, HL, and adoption of preventive behaviors in the intervention group than the control group (*P* < 0.05).

**Conclusion:**

The educational intervention based on an HBM integrated with HL was effective and acceptable in correcting and promoting walking and nutrition behaviors to prevent osteoporosis among health volunteers. Therefore, it can be said that the intervention implemented was in line with the developed model used.

## Introduction

Osteoporosis is the most common metabolic bone disorder characterized by a decrease in bone mass and disruption of the structural components of bone tissue [1]. Females are eight times more likely than males to develop osteoporosis [2]. In a systematic study in Iran, the prevalence of low bone density in females was reported 51% and the prevalence of osteoporosis 32%, which was 32% in the lumbar vertebrae, 21% in the spine, 25% in the neck, and 21% in the femur [3].

Osteoporosis leads to a wide range of complications, just as fracture pain that can last for a long time and cause loss of mobility [4]. Osteoporosis causes disability and disruption in people’s lives and causes many financial costs and physical disorders [5]. Prevention of osteoporosis has several aspects including nutrition, exercise, lifestyle, and initial screening. WHO believes that in the prevention of osteoporosis, individuals should be aware of a balanced diet containing vitamin D and calcium, and regular exercise [6].

Based on the results of researches on the behavior change, today successful preventive education is provided according to known models, and in this regard, the HBM in the prevention of diseases and behavioral problems is acceptable and effective [7]. In this model, the probability of the adoption of preventive behaviors is influenced by perceived threat, the self-efficacy, perceived barriers and benefits, and cues to action [7]. The HBM is especially useful for designing programs to prevent disease and change behavior in the short term [8].

**Fig. 1:**
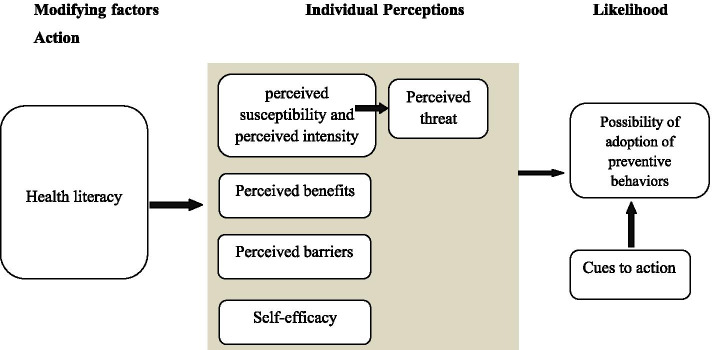
Integration of HL on HBM

The HL has a potential impact on the structures of the HBM [9, 10] and can be used as a moderating factor in the HBM instead of the awareness variable. This conceptual framework provides a useful tool for changing and interpreting the ways in which HL affects the rate of the desired behavior [10]. In addition, HL can indirectly influence osteoporosis preventive behaviors affecting the mentioned structures (Fig. 1).

The HL is one of the effective components in preventing osteoporosis among females [11]. HL plays an important role in attracting females to health-promoting activities and adoption of preventive behaviors, as well as in promoting their knowledge and ability to pursue a clinical care program [11]. According to the results of some studies, HL among women was at an inadequate or a borderline level [12, 13]. Studies have shown that low HL can be promoted by educating people [14, 15].

One of the important and basic conditions for the success of health programs is the participation of women, which means comprehensive involvement in the decision-making process and implementation in matters related to society. In this regard, the “health volunteers” project is underway. The purpose of this project, in addition to teaching the necessary materials and skills to the volunteers, is to convey health messages by them to the community [16].

The HBM has great potential in preventing osteoporosis [17–19] and promoting HL [9], in turn, HL plays a crucial role in preventing osteoporosis [11, 20], adoption of preventive behaviors [13, 21], and adoption of behaviors such as physical activity [22]. In addition, the use of the HL along with HBM, can facilitate and strengthen the performance of this model in adoption of preventive behaviors. Also, it can facilitate the use of this model to design educational interventions for people at any level of literacy (in other words, the educational intervention was commensurate with the level of literacy of the people).

In this study, this research question was raised whether the HBM integrated with HL can be effective in improving and promoting nutrition and walking behaviors by increasing HL or not?

The present study was performed among health volunteers aimed to determine the effect of educational intervention based on HBM integrated with HL on the adoption of walking and nutrition behaviors to prevent osteoporosis.

## Methods

This was a quasi-experimental, interventional study. The statistical population was active health volunteers referring to health centers in Qazvin, from which 140 people (70 people for each of the two intervention and control groups) were selected by multi-stage random sampling. In such a way that at first a list of health centers in Qazvin was prepared and then they were divided into two parts, north and south, based on the map. Then, from each part, two health centers (50%) were randomly selected for the intervention group and two health centers (50%) for the control group. In each health center (25%), health volunteers were randomly selected from a list of names through a lottery, the participation rate 94.59%.

Given the results of the study by Panahi et al. and considering P1 = 0.42 and P2 = 0.66 for the adoption of preventive behaviors (10), also using Pocock’s formula, considering the test power of 80% and statistical confidence interval of 95%, sample size for each group was estimated to be 64 people. Finally, for more accuracy and also to consider the 15% probability of sample drop, it was decided to consider 74 people for each group.

The inclusion criteria of the present study were literacy, Iranian citizenship, the age group ranging from 18 to 65 years, working as a health volunteer at the time of the study, active presence at the weekly or monthly meetings of volunteers in the health centers, providing informed written consent, and not attending osteoporosis training classes for the past 6 months. Furthermore, unwillingness to continue participating in the study, absence during the intervention, the history of participation in similar research, and incompletely answering the questionnaires were considered exclusion criteria.

A questionnaire consisting of five parts was used to collect data:

A) Demographic characteristics included questions about age, weight, height, marital status, level of education, employment status, weekly physical activity, monthly family income, smoking history, delivery history, breastfeeding history, family history of osteoporosis, bone density measurement history, the location of residence, and the number of family members.
B) The second part consisted of structural questions of the HBM. It included 4 questions to assess the perceived sensitivity of the standard scale (included the degree to which women found themselves at risk for osteoporosis), 6 questions for the perceived intensity of the standard scale (included questions about the side effects of osteoporosis), 8 questions for the perceived benefits of the standard scale (included questions about the benefits of osteoporosis preventive behaviors such as walking and calcium intake), 7 questions for the perceived barriers of the standard scale (included barriers to walking and eating calcium-containing foods), and 4 questions for self-efficacy (included the ability to exercise and follow a proper diet) [18]. The questions of the constructs of cues to action reviewed sources of information on osteoporosis health advice and were calculated as frequency measurement. It should be noted that in this study, the constructs of cues to action were used to determine the method of intervention according to the opinion of health volunteers [10]. All questions were scored on a 5-point Likert scale from “strongly disagree” to “strongly agree” (scores 1 to 5). The validity of the model constructs in the study of Jeihooni et al. was reported to be higher than 0.7. Moreover, the Cronbach’s alpha coefficient of the perceived sensitivity construct was 0.71, of the perceived intensity construct was 0.82, of the perceived benefits construct was 0.79, of the perceived barriers construct was 0.82, of the self-efficacy construct was 0.79, and of the whole instrument was estimated 0.87 [18]. In the present study, the Cronbach’s alpha coefficient for the perceived sensitivity construct was 0.79, for the perceived intensity construct was 0.86, for the perceived benefit construct was 0.78, for the perceived barrier construct was 0.84, for the perceived self-efficacy construct was 0.82, and for the whole questionnaire was calculated to be 0.83. It is worth noting that for questions of the cues to action, because it was in the form of objects and concrete and did not measure people’s perception, validity and reliability were not calculated [23, 24].
C) The third part was related to measuring awareness about osteoporosis and its complications and effects on individuals. The tool consisted of 23 questions, with two points for each correct answer, zero points for each incorrect answer, and one point for the “I do not know” option. The validity of the questionnaire was assessed by ten experts and the desired validity was obtained and Cronbach’s alpha was 0.92 [25]. In the present study, the reliability of the awareness variable through Cronbach’s alpha coefficient was estimated to be 0.82.
D) The fourth part was Health Literacy Instrument for Adults aged from 18 to 65 years [26]. The questionnaire consisted of 33 items in 5 domains of access (6 items), reading skills (4 items), comprehension (7 items), assessment (4 items), and decision making and behavior (12 items). The Scoring method was a 5-point Likert scale so that in the questions on reading skills the score 5 was assigned to the “quite easy” option, the score 4 to the “easy” option, the score of 3 to “neither easy nor difficult” option, the score of 2 to the “difficult” option, and the score 1 to the “completely difficult” option. Regarding other aspects of HL, the score of 5 was assigned to the “always” option, the score of 4 to the “most of the times” option, the score of 3 to the “sometimes” option, the score of 2 to the “rarely” option, and the score of 1 to the “never” (or “not at all”) option. The scoring method was such that the raw score of each person in each domain was obtained from the sum of scores. To convert this score to a range of 0 to 100, the formula for the difference of the raw score obtained from the minimum possible raw score divided by the difference of the maximum possible score of the minimum score was used. Then, to calculate the total score, the scores of all dimensions (based on the range from zero to 100) were added and divided by the number of dimensions (number 5). Scores from 0 to 50 were considered as inadequate HL, 50.1 to 66 as not so much adequate HL, 66.1 to 84 as adequate HL, and 84.1 to 100 as excellent HL [27]. This questionnaire was designed and analyzed psychometrically by Montazeri et al. [26] in Iran in 2014 and its validity and reliability have been confirmed. The construct validity was obtained desirable in 5 domains and in total 53.2%, the reliability of the instrument was obtained from 0.72 to 0.89 by determining the Cronbach’s alpha coefficient of items [26]. In the present study, the alpha coefficient for the reading domain was calculated 0.85, for the access domain was 0.82, for the comprehension domain was 0.79, for the assessment domain was 0.81, for the decision making and application of health information was 0.76, and for the whole HELIA questionnaire was 0.79.
E) The last part included measuring nutrition and walking behaviors to prevent osteoporosis. It consisted of 11 questions and assessed a person’s food intake, that was effective in preventing osteoporosis over the past week. The scoring method of this section was as follows: zero point was considered for not consuming the desired foods and one point was considered for consuming the desired foods. Thus, the range of scores was between zero and 11. The walking behavior section also included 7 questions and measured individuals’ walking duration over the past week according to the given guide. The scoring method in this section was as follows: zero point for “not walking”, 1 point for “light walking”, 2 points for “medium walking”, and 3 points for “heavy walking”. Thus, the scores ranged from zero to 21. The validity of nutrition and walking behavior questions in the study of Jeihooni et al. was more than 0.7 and the reliability of the instrument was determined higher than 0.79 by calculating Cronbach’s alpha coefficient [18, 28].

Data were collected by completing questionnaires in two stages, before and 3 months after the intervention. The control group only completed a questionnaire at the same time as the intervention group and did not receive any training. Participation in the study was voluntary and based on people’s willingness so that whenever they wanted to leave the study, there was no obligation to continue the study.

After collecting information in the pre-test stage, the members of the intervention group received e-learning through social media software, 4 times during 4 weeks (once a week) and were provided with educational booklets and pamphlets. The intervention was done through the social network (Telegram or WhatsApp), embedding a software download link in the social media environment, embedding website address and Telegram channel introduced in the social media environment, sending motivational messages to encourage participants, providing question and answer sessions in Cyberspace.

The content of training sessions for health volunteers included the definition of osteoporosis, causes and symptoms and risk factors, diagnosis, complications, healthy lifestyle, various strategies to prevent osteoporosis, the role of nutrition in preventing osteoporosis, dietary benefits and barriers, following dietary recommendations, self-efficacy in following a proper diet, and the role of exercise and walking in preventing osteoporosis. In each training session, in addition to training, items such as Q&A cases and bug fixes were used. At the end of each session, the related training booklets were given to the individuals. Researchers were present at the sessions to conduct and monitor the implementation of the sessions. To facilitate the educational process, an educational manual was used, which was prepared by the researcher based on up-to-date books and scientific resources and an educational PowerPoint (Table 1).

**Table 1:** Training sessions

Session number	Educational content	Time sessions
Session 1	Introducing the researcher and colleagues and expressing the educational goals; starting the training about the definition of osteoporosis, causes and symptoms and risk factors, diagnosis, complications.	90 min
Session 2	Healthy lifestyle; various strategies to prevent osteoporosis; the role of nutrition in preventing osteoporosis.	80 min
Session 3	Dietary benefits and barriers; following dietary recommendations.	90 min
Session 4	Self-efficacy in following a proper diet.	90 min
Session 5	Role of exercise and walking in preventing osteoporosis.	90 min
Session 6	How to calculate the amount of drug used and the times of its use; explain the concept of BMI and how to measure it.	80 min
Session 7	The importance of proper use and storage of drugs; the importance of preventing chronic diseases.	90 min
Session 8	The importance of using reliable sources in obtaining health information.	90 min
Session 9	Summarizing all the training materials and reviewing them and answering patients’ questions.	80 min

Regarding the ethical considerations in this research, first, the related project code was received from the Vice-Chancellor for Research and Technology of Qazvin University of Medical Sciences (with the ethics code number IR.QUMS.REC.1398.380) and the necessary coordination was done with the selected health centers. Besides, the purpose of this study was explained to health volunteers and their written consent was obtained. It was then informed that the inclusion of these individuals in the study was entirely voluntary and their anonymity was emphasized to ensure that the information would be collected and kept confidential. The self-reported method was used in completing the questionnaires.

All methods were carried out in accordance with relevant guidelines and regulations. All experimental protocols were approved by a named institutional and/or licensing committee. In addition, written informed consent from all the participants were obtained and they were granted the right to withdraw from the study at any time. After collection, the data were entered into SPSS 23. In data analysis, first, using the Kolmogorov-Smirnov test, the data distribution status was examined in terms of normality and non-normality. The results of the Kolmogorov-Smirnov test showed that the distribution of data in the variables of awareness, HL, and all structures of the HBM was non-normal and in the case of behavioral variables was normal. Then a test fit the data was performed. A paired t-test (for normal data) and in some cases, the non-parametric Wilcoxon test (for non-normal data) were used to examine the groups for changes in the dependent variable. To compare the intervention group with the control group, for quantitative variables, the independent t-test (for normal data) and in some cases, the non-parametric Mann-Whitney test (for non-normal data) were used and to evaluate the qualitative variables, chi-square and when necessary Fisher’s exact tests were used. Meantime, the significance level in this study was considered 0.05.

## Results

Totally 70 health volunteers in the intervention group and 70 health volunteers in the control group completed the study course (8 participants were excluded from the study due to incompletely answering the questionnaires). Then, statistical analyzes were performed on the participants that according to the results, there was no significant statistical difference between the intervention and control groups in terms of demographic and background variables (*P* > 0.05). In other words, the two groups were homogeneous in terms of demographic and background characteristics (Table 2). Moreover, the results of the t-test revealed that there was no significant statistical difference between the mean age of health volunteers in the intervention and control groups (*P* = 0.619). The mean and standard deviation of the age of the health volunteers in the intervention and control groups were 41.24 ± 3.48 and 40.18 ± 3.58 years, respectively.

**Table 2:** Demographic and background characteristics related to health volunteers of the intervention and control groups

Demographic and background variables		Intervention group		Control group		***P***-value
		Numbers	Percentage	Numbers	Percentage	
**Weight (KG)**	Less than 60	29	41.4	31	44.3	0.312**
	60 to 80	37	52.9	36	51.4	
	More than 80	4	5.7	3	4.3	
**Height (CM)**	Less than 160	29	41.4	32	45.7	0.413*
	More than 160	41	58.6	38	54.3	
**Marital status**	Single	10	14.3	11	15.7	0.213**
	Married	57	81.4	55	78.6	
	Divorce or spouse’s death	3	4.3	4	5.7	
**Level of education**	Under diploma	15	21.5	13	18.6	0.178*
	Diploma	37	52.8	40	57.1	
	Post-diploma and bachelor’s degree	18	25.7	17	24.3	
**Employment status**	Housekeeper	48	68.6	47	67.1	0.557**
	Employee	8	11.4	11	15.7	
	Self-employment	4	5.7	2	2.9	
	Other	10	14.3	10	14.3	
**Physical activity per week**	Every day	10	14.3	11	15.7	0.145*
	Most of days	11	15.7	14	20	
	Sometimes	21	30	18	25.7	
	Rarely	14	20	12	17.1	
	Never	14	20	15	21.5	
**Monthly income of the family**	Less than 2 million	13	18.6	16	22.9	0.098*
	2 to 3 million	38	54.3	36	51.4	
	More than 3 million	19	27.1	18	25.7	
**History of smoking**	Yes	4	5.7	1	1.5	0.316**
	No	66	94.3	69	98.5	
**History of labor**	Yes	60	85.7	59	84.3	0.455*
	No	10	14.3	11	15.7	
**Breastfeeding history**	Yes	55	78.6	51	72.9	0.288*
	No	15	21.4	19	27.1	
**History of osteoporosis of family**	Yes	3	4.3	5	7.1	0.219**
	No	67	95.7	65	92.9	
**History of bone density measurement**	Yes	14	20	11	15.7	0.186*
	No	56	80	59	84.3	
**The location of residence**	Qazvin	49	70	51	9/72	0.255*
	Counties around Qazvin	15	21.4	14	20	
	Village	6	8.6	5	7.1	
**The number of family members**	3 people	34	48.6	38	54.3	0.121*
	4 people	27	38.5	25	35.7	
	5 people and more	9	12.9	7	10	

*Chi-square test (significant correlation less than 0.05)

**Fisher’s exact test

The results showed that before the intervention, there was no significant difference between the mean scores of the variables of awareness, all constructs of the health belief model, and HL in the two groups of intervention and control, based on the Mann-Whitney test. Three months after the intervention, there was a statistically significant difference between the mean scores of the variables of awareness, all constructs of the health belief model, and HL in the two groups. In addition, the results of the Wilcoxon test stated that in the intervention group, there was a statistically significant difference between the mean scores of the variables of awareness, all constructs of the health belief model, and HL, before and after the intervention (*P* < 0.05). While in the control group in this regard in the two stages, before and after the intervention, no statistically significant difference was observed (*P* > 0.05). The results also indicated that before the intervention, there was no significant difference between the mean scores of the variables of adoption of preventive behaviors such as nutrition and walking in the intervention and control groups, based on the independent t-test. Three months after the intervention, there was a statistical significant difference between the mean scores of the variables of the adoption of preventive behaviors like nutrition and walking in the two groups. Moreover, the results of the paired t-test stated that in the intervention group, there was a statistical significant difference between the mean scores of the variables of the preventive behaviors adoption such as nutrition and walking, before and after the intervention (*P* < 0.05). While in the control group in this regard, before and after the intervention, no statistical significant difference was observed (*P* > 0.05) (Table 3).

**Table 3:** Comparison of the mean scores of awareness, HBM constructs, HL, and adoption of preventive behaviors (walking and nutrition) of osteoporosis during the study course in two intervention and control groups consisting health volunteers

Constructs and variables	Time	Before intervention		3 months after intervention		***P***-value *
	Groups	Mean	Standard deviation	Mean	Standard deviation	
**Awareness**	Intervention	**27.113**	**1.141**	**39.233**	**1.115**	**< 0.001**
	Control	**26.972**	**1.337**	**28.184**	**1.144**	**0.131**
*P*-value**		**0.557**		**< 0.001**		
**Perceived intensity**	Intervention	**15.251**	**0.491**	**20.442**	**0.409**	**< 0.001**
	Control	**15.123**	**0.542**	**15.660**	**0.513**	**0.259**
*P*-value**		**0.716**		**< 0.001**		
**Perceived sensitivity**	Intervention	**13.423**	**0.489**	**17.257**	**0.335**	**< 0.001**
	Control	**14.117**	**0.431**	**15.025**	**0.314**	**0.217**
*P*-value**		**0.354**		**0.011**		
**Perceived benefits**	Intervention	**24.993**	**0.788**	**31.224**	**0.416**	**< 0.001**
	Control	**25.119**	**0.414**	**25.557**	**0.313**	**0.417**
*P*-value**		**0.322**		**< 0.001**		
**Perceived barriers**	Intervention	**20.058**	**0.119**	**23.112**	**0.334**	**0.003**
	Control	**19.817**	**0.214**	**20.055**	**0.499**	**0.158**
*P*-value**		**0.155**		**0.004**		
**Self-efficacy**	Intervention	**11.558**	**0.654**	**17.428**	**0.425**	**< 0.001**
	Control	**11.665**	**0.547**	**12.018**	**0.561**	**0.119**
*P*-value**		**0.387**		**< 0.001**		
**Health literacy**	Intervention	**59.898**	**5.122**	**71.411**	**6.544**	**0.003**
	Control	**60.585**	**5.255**	**62.017**	**6.414**	**0.279**
*P*-value**		**0.314**		**< 0.001**		
**Nutrition behaviors**	Intervention	**5.398**	**1.447**	**8.466**	**1.244**	**0.009**
	Control	**5.451**	**1.222**	**6.003**	**1.005**	**0.351**
*P*-value**		**0.543**		**< 0.001**		
**Walking behaviors**	Intervention	**8.956**	**0.261**	**13.457**	**0.587**	**< 0.001**
	Control	**8.848**	**0.353**	**9.025**	**0.545**	**0.211**
*P*-value**		**0.414**		**< 0.001**		

*Mean changes during the course, separately in each group

**Comparing the differences between the two groups

## Discussion

This study aimed to determine the effect of educational intervention based on the HBM integrated with HL on the adoption of walking and nutrition behaviors to prevent osteoporosis among health volunteers. In the present study, after the implementation of the educational intervention, the mean score of awareness increased significantly in the intervention group and a significant difference was observed between the two groups in terms of mean score of awareness. This result revealed that education had a positive effect on raising the awareness of the health volunteers about osteoporosis. Possible reasons for the increase in awareness scores in the intervention group can be as following: the desire of health volunteers to play an active role in promoting the health of themselves, family and neighbors [29, 30]; complete educational intervention in this study; use different methods to implement the intervention; provide Q&A during the sessions as well as in the cyberspace; receive educational packages and pamphlets at the end of each session of the intervention; and also to send short messages to encourage participants during the follow-up period. Furthermore, considering the significant relationship between awareness and HL [31, 32] and also the role of HL in completing people’s awareness in the HBM [33], it can be said that one of the reasons for increasing the health score of health volunteers in the intervention group, was increasing their HL. These results were in line with the results of studies by Parandeh et al. [34], Khani Jeihooni et al. [28], Sanaeinasab et al. [35], and Ghaffari et al. [36].

The results of the present study indicated that after the intervention, the mean score of perceived intensity in the test group increased significantly compared to the control group. Perceived intensity depended to some extent on individuals’ awareness and had a strong relationship with it [37]. Therefore, it can be said that increasing awareness has led to an increase in people’s perceptions of the intensity of osteoporosis and its complications. Another possible reason for the increase in perceived intensity could be an increase in the HL of the health volunteers in the intervention group. This finding was consistent with the results of studies by Karimi Aval et al. [38] and Hazavehei et al. [39].

Based on the results of the study in the intervention group, the mean score of perceived sensitivity increased significantly after education and there was a significant difference between the two groups in this regard. Perceived sensitivity was considered as one of the influential factors in the adoption of preventive behaviors, and real and successful prevention depended on real information about personal sensitivity and related risks [25]. Perceived sensitivity, like perceived intensity, had a strong cognitive component and was somewhat dependent on individuals’ awareness [40]. Therefore, the education has probably been able to increase the perceived sensitivity in health volunteers by increasing the level of awareness and information about the risks of osteoporosis. As a result, they have found themselves at risk for osteoporosis more than those of the control group. Another probable reason for perceived sensitivity increment could be HL increment and thus creating sufficient perceived sensitivity [33]. This result was consistent with the results of studies by Parandeh et al. [34], Jeihooni Khani et al. [28], Karimi Aval et al. [38], Sanaeinasab et al. [35], and Shojaeizadeh et al. [25].

In the present study, after the implementation of the educational intervention, the mean score of perceived benefits increased significantly in the test group and a significant difference was observed between the two groups in this regard. One of the reasons for increasing the score of perceived benefits could be the reduction of perceived barriers in this study [38]. Besides, the educational intervention may has been able to determine the benefits of adoption of osteoporosis preventive behaviors more than before for the people in the intervention group by increasing awareness and HL. This result was consistent with the results of studies by Karimi Aval et al. [38] and Khorsandi et al. [40].

Based on the results, after the intervention, there was a significant difference between the two groups in terms of the mean score of perceived barriers. One reason for the decrease in perceived barriers scores could be the increase in perceived benefits in this study [38]. Similarly, increasing perceived intensity could indirectly reduce perceived barriers [38]. Another reason for decreasing the score of perceived barriers in the test group in this study could be to increase self-efficacy because self-efficacy affects perceived barriers and higher self-efficacy reduces perceived barriers in performing the behavior [41]. The HL could also be effective in reducing perceived barriers by creating sufficient awareness and increasing perceived sensitivity, perceived intensity, perceived benefits, and self-efficacy [42]. This result was consistent with the results of studies by Jeihooni Khani et al. [28] and Karimi Aval et al. [38].

In the present study, after the implementation of the educational intervention, the mean score of self-efficacy increased significantly in the test group and a significant difference was observed between the two groups in this regard. Hence, it can be said that people who have more self-efficacy, consider higher goals and would become more committed, and as a result, their behavior would become more desirable. Increasing self-efficacy of the intervention group in this study could be due to increasing the level of HL because increasing HL has a positive effect on improving self-efficacy [10, 43]. Other possible reasons for self-efficacy increment could be perceived barrier reduction [42] and awareness increment. This result was in line with the results of studies by Karimi Aval et al. [38], Jeihooni et al. [28], and Torbaghan et al. [44].

Based on the results in the intervention group, after education, the mean scores of HL increased significantly and there was a significant difference between the two groups in terms of mean HL. Regarding the increase of HL in this study, it can be said that one of the roles of HL among the constructs of the HBM is to create full awareness and adequate perceived sensitivity [32]. Therefore, it can be concluded that educational intervention has been able to improve people’s sensitivity by increasing awareness. As a result, people in the intervention group probably paid more attention to key points than before, including disease symptoms, when to see a doctor, how often to check for symptoms, how often to have periodic checkups (Check-up), how to store and take medications, how to calculate body mass index and maintain it at normal levels, and things like that. Subsequently, these cases have been able to increase their HL. Other possible reasons for increasing HL included increasing people’s awareness and self-efficacy because HL is directly related to both awareness [29–31] and self-efficacy [10]. The results of this section were consistent with the results of studies by Panahi et al. [10], Panahi et al. [45], Ebrahimpour et al. [46], and Zhuang et al. [47].

In the present study, after the implementation of the educational intervention, the mean score of nutrition and walking behaviors to prevent osteoporosis increased significantly in the test group and a significant difference was observed between the two groups in this regard. The results of several studies were consistent with the results obtained in this section [28, 48]. The results of the present study revealed that the educational intervention based on the HBM integrated with HL was able to promote the adoption of preventive behaviors by increasing awareness and HL and changing the constructs of the HBM (people’s attitudes). Hence, it can be said that the intervention performed was appropriate to the integrated model used. Consistent with the present study, the study of Panahi et al., the implementation of an educational intervention based on the HBM integrated with HL, led to the promotion of preventive behaviors [10].

The most important limitation of this study was the lack of specific tools for measuring HL in the field of osteoporosis. The target group in this study were active health volunteers, resident in Qazvin province. Therefore, the results of this study cannot be generalized to other groups of health volunteers in other cities. Since, performing this study is recommended among health volunteers in other cities as well as among different groups of women (in terms of education level, age, and region of residence). The use of self-report method to measure participants’ behavior was another major limitation of this study. Meanwhile, the researchers were unable to assess the impact of the education provided to health volunteers on the families covered by them, which could be the title of the research team’s next study. The advantages of this plan can be designing the intervention based on an integrated model consisting of the structures of the HBM and HL; paying attention to the media interests of all subjects; paying attention to the level of HL of the subjects and designing an educational program according to their level of HL; as well as the adaptation of the research topic to the current needs of the health system; and the implementation of the study among health volunteers, which could pave the way for change in the families covered by health volunteers.

## Conclusion

In general, the results of the present study revealed that the educational intervention based on the HBM and HL, could increase awareness, change attitudes, increase HL and adoption of nutrition and walking behaviors to prevent osteoporosis among health volunteers. Therefore, it can be said that the intervention implemented was in line with the developed model used.

## Data Availability

The data that support the results of this study are available by [Leila Dehghankar] but there are restrictions on the availability of this data, which were used under license for the current study, and so are not publicly available. Data are however available from the authors upon reasonable request and with permission of [Leila Dehghankar].
